# Lipid Nanotechnology

**DOI:** 10.3390/ijms14024242

**Published:** 2013-02-21

**Authors:** Samaneh Mashaghi, Tayebeh Jadidi, Gijsje Koenderink, Alireza Mashaghi

**Affiliations:** 1 Zernike Institute for Advanced Materials, Centre for Synthetic Biology, Nijenborgh 4, 9747 AG Groningen, The Netherlands; E-Mail: s.mashaghi@rug.nl; 2 Department of Physics, University of Osnabrück, Barbarastraße 7, 49076 Osnabrück, Germany; E-Mail: tayebeh.jadidi@uos.de; 3 FOM Institute AMOLF, Science Park 104, 1098XG Amsterdam, The Netherlands; E-Mail: g.koenderink@amolf.nl; 4 Department of Bionanoscience, Kavli Institute of Nanoscience, Delft University of Technology, Lorentzweg 1, 2628 CJ Delft, The Netherlands

**Keywords:** soft nanotechnology, supramolecular chemistry, hybrid materials, synthetic biology, self-assembly, nanoparticle, nanomedicine, nanofluidics, nanoelectronics, lab-on-a-chip

## Abstract

Nanotechnology is a multidisciplinary field that covers a vast and diverse array of devices and machines derived from engineering, physics, materials science, chemistry and biology. These devices have found applications in biomedical sciences, such as targeted drug delivery, bio-imaging, sensing and diagnosis of pathologies at early stages. In these applications, nano-devices typically interface with the plasma membrane of cells. On the other hand, naturally occurring nanostructures in biology have been a source of inspiration for new nanotechnological designs and hybrid nanostructures made of biological and non-biological, organic and inorganic building blocks. Lipids, with their amphiphilicity, diversity of head and tail chemistry, and antifouling properties that block nonspecific binding to lipid-coated surfaces, provide a powerful toolbox for nanotechnology. This review discusses the progress in the emerging field of lipid nanotechnology.

## 1. Introduction

Nanotechnology involves fabrication of nano-devices with length scales of the order of 100 nm or smaller. Traditionally, nano-devices were made of metals, ceramics and inorganic semiconductors [1]. However, nano-devices can also be made of organic polymers, colloids or biomolecules, including DNA, proteins and lipids. This alternative approach is known as soft nanotechnology [2]. Soft nanotechnology has brought new concepts to electronics, medicine and the energy industry, complementing hard nanotechnological approaches. Examples include step-and-flash imprint lithography, phase-shifting lithography using elastomeric masks, and chemical-mechanical polishing [3–5]. Moreover, development of novel nanoscale targeting approaches for imaging and drug delivery has brought new hope to cancer and cardiac patients [6,7].

Lipids have a range of desirable properties for use in nanotechnology. Lipids can self-assemble into nano-films and other nano-structures such as micelles, reverse micelles and liposomes [8]. While lipid molecules self-interact to make nanostructures, the resulting structures display virtually no non-specific binding of other biomolecules, a property which allows lipid-based nano-devices to function within the human body. Lipid assemblies may, however, be glued to other soft or hard nano-devices via specific chemical linkages. These features, along with transparency of lipid structures in visible light and their heat conductivity, have made lipids an important soft matter building block for nanotechnology.

Hard and soft nano-devices for medical applications typically interface lipids while in use. Sensitive sensing platforms are for instance being developed that allow high-throughput sensing of biomolecular processes, building on advances in nano- and microfabrication and in nanophotonics. These sensing devices often include lipid molecules and lipid assemblies. Another device category are drug nanocarriers that encounter numerous barriers *en route* to their target, such as mucosal barriers and cellular membranes, both containing a repertoire of phospholipids and other lipid molecules [9]. Therefore, understanding the interactions between lipids and these nano-devices is essential for the optimal design of the nano-devices.

Computational tools have become increasingly important in lipid nanotechnology and nanotechnology in general. Over the last two decades, computational nanotechnology resulted in novel concepts and designs which were later realized or verified through experiments [10,11]. Computational nanotechnology provides a convenient way to explore the wide range of possible designs, allowing rapid evaluation and elimination of obvious dead ends and screening for more promising designs [11,12]. With regards to nanomedicine, computational nanotechnology has been widely used to help develop our understanding of the possible hazards associated with newly developed nano-products and to find practical solutions [13,14].

Despite the widespread use of lipids in nanotechnology, lipid nanotechnology has not yet been recognized as a field. Selected topics in lipid nanotechnology have been reviewed elsewhere, for instance in the area of nano-structured sensing platforms for membrane processes and lipid nanotechnology in food science [15]. Here, we aim to present the state-of the-art in experimental lipid nanotechnology with particular focus on selected areas that have not been reviewed elsewhere, yet hold great promise. Moreover, we discuss how computational approaches in the past few years contributed to this field. Finally, we identify key unresolved challenges that lie ahead.

## 2. Experimental Lipid Nanotechnology

**Figure 1:**
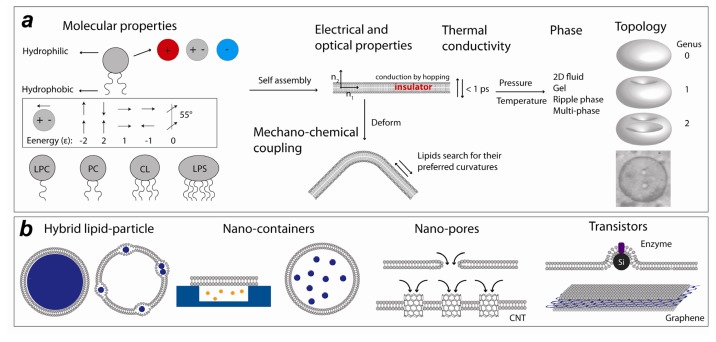

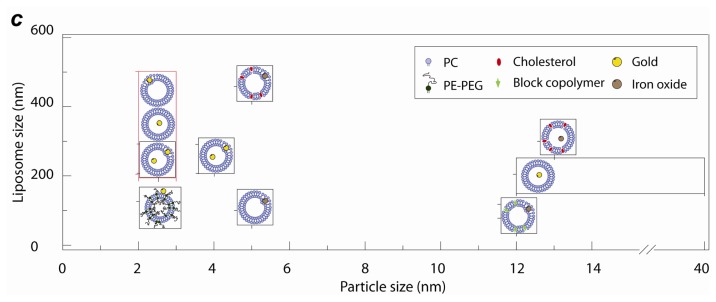
(**a**) Physico-chemical properties of phospholipids. Phospholipids are composed of a hydrophilic head group and hydrophobic tails (*N* = 1–6). PC: phosphatidylcholine, LPC: LysoPC, CL: cardiolipin, LPS: lipopolysaccharide. The head group may carry a positive or negative charge. It may also be neutral but possess a significant electric dipole which enforces particular arrangements in the monolayer and bilayer assemblies. Self assembly of phospholipids results in bilayers or monolayers. Bilayers are birefringent [28], thermally conductive (energy transfer on sub-picosecond time scales; κ~several Watt/mK [20]), behave as insulators perpendicular to the membrane and in the core part whereas they are electrically conductive within the plane of the membrane due to proton hopping in the interfacial water [29]. Assembled bilayers display different phases depending on the temperature (*T*) and pressure (*P*) of the environment. The fluid bilayer is also deformable and introducing curvature may lead to spatial redistribution of lipids, some preferring certain curved regions. Lipid bilayer can form topologically rich structures: a spherical bilayer (genus 0) vesicle, a genus 1 and a genus 2 vesicle [17,19] are shown. In addition to phospholipids, also other lipids such as sterols have been used in nanotechnological applications; (**b**) Lipid based nano-devices. Two examples of hybrid lipid particles are presented: in one case the particles are enclosed by the vesicle and in the other case the particles are embedded in between the leaflets. Nano-containers can be formed when a hard nano well is sealed by a lipid bilayer. Membrane pores can be generated within a bilayer by applying voltages or with the help of carbon nanotubes functionalized with hydrophilic groups at their ends. A composite structure made of silicon nano-wire and enzyme functionalized lipid bilayer is shown. Another example of lipid based nanostructures with applications in electronics is lipid bilayer-graphene hybrid where graphene is embedded in between the leaflets of a bilayer and thereby the graphene’s physical properties are modulated; (**c**) Liposomes functionalized with nanoparticles allowing for radiation/magnetic field triggered cargo release [30–36]. The ticks on the box sides indicate the size or the size range of the synthesized particles.

Phospholipids—the dominant lipids in biomembranes—are amphiphilic molecules with hydrophobic tails and hydrophilic head groups (Figure 1a). The head groups can be charged (positively or negatively) or neutral. Phosphatidylcholine (PC)—the most prevalent phospholipid—is a zwitterionic lipid with a significant electric dipole, which governs inter-lipid interactions [16,17]. Hydrophobic interactions drive spontaneous assembly of lipids in an aqueous environment into lipid bilayers (see also BOX I for advances in lipid bilayer engineering). Bilayers are quasi-2D (4 nm thick) nano-films which show rich phase behavior depending on temperature and pressure. Bilayers can be in fluid, gel, interdigitated and ripple phases. Phospholipids vary in their melting temperatures and influence the melting of the neighboring lipids in the bilayer in a cooperative manner. For bilayers with mixed compositions, at certain temperatures and pressures, different bilayer phases may co-exist [18].

BOX I **Advances in lipid bilayer engineering**

**(1) Solid-supported lipid bilayer**

Multiple strategies exist for deposition of a thin organic film on a solid substrate, including thermal evaporation, sputtering, electrodeposition, molecular beam epitaxy, adsorption from solution, the Langmuir-Blodgett technique, self-assembly, *etc.* [53]. Supported lipid bilayers (SLB) are commonly prepared [54] by three different strategies; the deposition of Langmuir monolayers, rupture of liposomes deposited on a surface, and self-assembly of spin-coated lipids upon hydration [55,56].

The Langmuir-Blodgett (LB) technique enables homogeneous deposition of the monolayer and bilayer over large areas with the possibility of making multilayer structures with varying layer composition [53]. This technique can be applied to almost any kind of solid substrate. SLBs can be formed by self assembly of liposomes on hydrophilic surfaces (glass, silica, titania or mica), hydrophobic surfaces (sialinated glass) or metals (using alkanethiols or thiolipid) [57–61]. The kinetics of the self-assembly reactions strongly depends on the composition and size of the liposomes, the chemistry and roughness of the surface and the buffer conditions [62–64]. A combination of LB monolayer transfer and liposome fusion can also be used to generate SLB [65]. In this method, liposomes are fused to a predeposited monolayer of phospholipid. The method allows formation of asymmetric bilayers [66].

**(2) Polymer-supported lipid bilayer**

The distance between the SLBs and the underlying supports is typically 0.2–1 nm, which means there is hardly any free volume underneath the lipid layer [67,68]. This hampers inserting protein, studying membrane transport processes, and establishing electrochemical gradients across the bilayer. Strategies have been proposed to increase the distance between the bilayer and the solid support: the use of polymer cushions, spacer lipids, and surfaces patterned with different thiol compounds.

Various polymeric films have been used as cushions for SLB. Polyethylene glycol (PEG), cellulose and dextran are among the most commonly used polymers for making polymer cushioned SLB [69–74]. A hydrated polymer cushion acts as a spacer and a lubricating layer between the lipid bilayer and the substrate and assists self-healing of local membrane defects [75,76]. In addition to a cushioning polymer film, alternative strategies include self-assembled monolayers (SAMs) [77,78] and the use of adsorbed or bound proteins as a cushioning layer [79–83]. One could also directly tether the membrane to a lipid presenting polymer or peptide layer [68,84,85]. One approach is to use synthetic lipids with their head group linked to a polymer that provides a cushion (most commonly PEG). Both the liposome fusion [86] and the spin coating method were successfully applied to form such polymer-cushioned bilayers [74,86]. Fusion of liposomes containing PEG-lipids gets kinetically slowed down when the density and molecular weight of the PEG molecules are increased [87–89]. Recently, PEG-SLBs with high density PEG spacers were successfully prepared by one of us when it was observed that addition of ethylenediaminetetraacetic acid disodium salt dihydrate (EDTA) promotes fusion of PEG-liposomes to form a PEG-SLB in the polymer brush regime. PEG interacts only weakly with commonly used substrates such as silica at neutral pH. Addition of 20 mM EDTA to the liposome solution (in TBS pH 7.4) shortly before exposing the solid support to the liposome solution results in a drop of pH and allows for the formation of, for example, PEG-SLBs with POPC/DOPE-PEG(2k) and POPC/DOPE-PEG(5k) with a DOPE-PEG molar percentage well into the brush regime. EDTA can be then washed away without affecting the integrity and fluidity of the bilayer.

**(3) Pore-spanning lipid bilayer**

Black lipid membranes (BLM) are widely used to form lipid bilayers spanning across apertures that are several hundreds of micrometers wide [90]. One of the issues with BLM is that they have a short lifetime. Fortunately the lifetime of the BLM significantly increases as the size of the aperture is reduced down to hundreds of nanometers [91]. Recently there have been many efforts to prepare BLM on nano-sized pores [92–94]. BLM are often prepared by painting, but alternatively, liposome fusion. The main challenge is to detect the formation of bilayer over the pores. The existing approaches are either demanding, slow and invasive or lack the desired detection sensitivity. AFM and Stimulated Emission and Depletion Imaging (STED) have recently been used to detect and characterize nano-pore spanning bilayers [95,96].

**(4) Protein incorporation into membranes**

A method that is conventionally used to insert trans-membrane proteins into supported lipid bilayers is to prepare proteoliposomes and prepare SLB by liposome fusion [65,68]. This is practically very difficult: there is no control on the orientation of the protein and the success of the method is limited to certain lipid compositions. Furthermore the narrow spacing between the SLB and the support hinders incorporation of large proteins such as cellular adhesion proteins that may have intracellular domains larger than 10 nm. This problem can in principle be overcome by making polymer-cushioned SLB or alternatively by nano-pore spanning bilayers. This is an active area of research and for certain proteins, incorporation into bilayers [97,98] and controlled redistribution of incorporated proteins [99] have already been successfully achieved.

Fluid membranes can be bent to make curved membranes for example by deforming the support of a supported lipid bilayer (SLB), by application of mechanical force onto the plasma membrane and also by spontaneous vesicle formation in an aqueous solution (Figure 1a). Inducing curvature has two consequences: (1) leads to lipid redistribution and (2) permeabilizes the membrane at the highly curved bending sites. The geometrical shape of phospholipids defines their preferred membrane curvature. Natural phospholipids often have two tails and have a nearly cylindrical shape, which favors formation of flat bilayers. However, some lipids favor high curvature, such as cardiolipin, which has four tails, and lysoPC, which has only one tail. Bilayers tend to close spontaneously into spherical (genus 0) objects called vesicles or liposomes. Liposomes can be made as small as a few tens of nanometers in diameter. Bilayers can also be used to make more topologically complex structures such as bilayer-based genus 1 and genus 2 objects [19] (objects with *g* number of holes are called genus *g*).

Phospholipid bilayers are aligned molecular systems with interesting thermal, optical and electronic properties [20–24] (Figure 1a). With respect to electrical conductance, the hydrocarbon tail region is an insulator, but significant conduction occurs at the interfacial water due to proton hopping [24]. Lipid bilayers also allow ion transfer when nano-pores are inserted [25]. The tails of the common phosphpolipids in the bilayer show significant alignment. Aligned polymer systems have recently been found to show very high thermal conductivities [26]. It has been found that polyethylene, which is an insulator in bulk, becomes a conductive material when the polymer molecules are aligned. Recent studies have demonstrated efficient transfer of vibrational energy along single lipid molecules [20] and monolayers [27] which suggests a significant thermal conductivity for lipid bilayers. These properties, along with antifouling (passivation), self-healing, and the specific optical properties (such as birefringence) lipid bilayers, make the phospholipids a very attractive soft matter material to nanotechnologists (Figure 1b,c). Several areas in which lipids have been used to make nano-size devices are discussed below.

### 2.1. Lipid Nanomedicine

**Figure 2:**
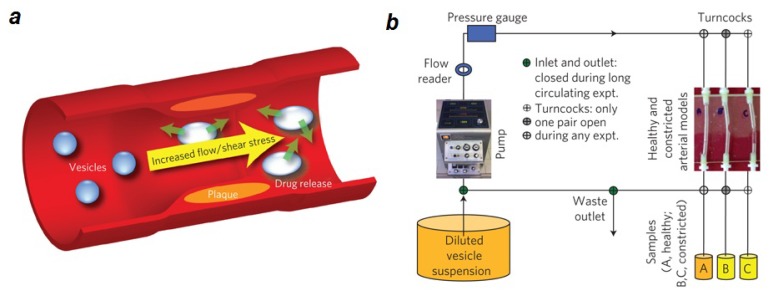

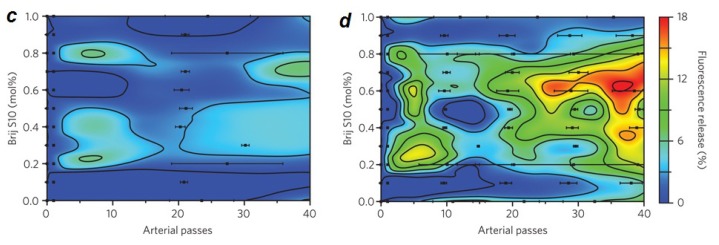
Shear-stress sensitive nano-sized lipid vesicles for targeted drug delivery. (**a**) Schematic of the design, in which changes in endogenous shear stress trigger drug release from the vesicles. (**b**) Experimental set-up. An extracorporeal heart pump is connected to a plastic mimic of normal or constricted arteries and the loaded vesicles are allowed to circulate in the artificial cardio-vascular system for 20 min. (**c**,**d**) Fluorescence release patterns of PC vesicles with 0–1 mol% Brij S10 at 37 8 °C. Release in the normal arterial model (**c**) and in the constricted artery model (**d**). Brij S10 concentration is plotted *versus* number of passes through the vascular system, with fluorescence release along the z-axis. After subtraction of background fluorescence release, the fluorescence signal represents the fractional additional release as result of circulation in the arterial model. Figure is adapted from [46] with permission.

Lipids often appear in nanomedical products, particularly in gene and drug delivery technologies. When DNA/RNA molecules or drugs are enclosed in a lipid-based container, they last longer with lower degradation rates compared to molecules in solution [37]. Such enclosure also increases the chance for endocytosis and uptake of DNA/RNA or drugs by cells [38–42]. Another important usage of lipids in constructing nanoparticles and nanocontainers has been to increase the targeting specificity, transport efficiency and potency of drugs. For example, the targeting specificity of porous nanoparticles was increased by designing porous nanoparticle-supported lipid bilayers (protocells). When these nanoparticles were functionalized with a targeting peptide for human hepatocellular carcinoma, a 10,000-fold greater affinity for human hepatocellular carcinoma than for hepatocytes, endothelial cells or immune cells was achieved (10^6^-fold improvement over comparable liposomes) [41,43]. Unprecedented specificity in binding to cancer cells has also been reported for similar designs of functionalized lipid-membrane coated silica nanoparticles [44]. The lipid bilayer on the nanoporous supports shows an increased fluidity (compared to e.g., liposomes), which enables selective targeting at low peptide densities. Moreover, lipid membranes prevent nonspecific binding of the nanoparticles, thus further enhancing the targeting specificity. This increased specificity has brought new hope for preventing drug resistance in cancer therapy [45]. Drug-loaded vesicles with lenticular morphology have been designed to specifically target atherosclerotic sites in blood vessels. In these locations, shear stress induces instabilities along the equator of the vesicles and drug release [46] (Figure 2).

An example of a case where usage of lipids led to an increase in molecular passage through the plasma membrane is DNA delivery by lipid-coated nano-calcium-phosphate (LNCP). While DNA delivery by calcium-phosphate nano-particles [47] has low and irreproducible transfection efficiency relative to other non-viral approaches (such as liposomes and polymers), DNA delivery by LNCP was found to be 24 times more efficient than the naked pDNA and 10-times more efficient relative to the widely used colloidal calcium-phosphate precipitation preparations [48]. Similarly, lipid-polymer composites for carrying DNA/RNA with high transfection efficiency have been designed and characterized [49]. An improved uptake rate has also been reported for cancer drug delivery. Supported lipid bilayers (SLB) were deposited on functionalized colloidal mesoporous silica (CMS) nanoparticles to fabricate a core-shell hybrid system (SLB@CMS). The SLB encapsulates the loaded molecules (e.g., drugs) and prevents their escape. The anticancer drug colchicine was loaded onto SLB@CMS particles, which were readily taken up by cells, leading to the depolymerization of microtubules with surprisingly enhanced efficiency as compared to the equal dose of drug in solution [50]. Finally curved membranes show increased affinity to peptides and proteins with curvature sensing capabilities [51].

Hybrid lipid nano-containers not only improve targeting in drug therapy, but also influence the type of the response elicited by the drug. For example, while bolus delivery of soluble peptide vaccines led to weak humoral responses with predominantly IgG1 production, lipid/polymer based nanocapsules containing the peptide promoted robust antigen-specific humoral immune responses with a balanced generation of multiple IgG isotypes [52].

### 2.2. Lipid Nanofluidics

The combination of micro-nanofluidics and lipid science has benefited both fields. On the one hand, microfluidics has been used to produce liposomes with diameters of the order of tens of nanometers as well as giant vesicles [100–103]. On the other hand, lipids have been used for passivation of fluidic channels, for material transport within fluid environments and for droplet engineering.

By controlled microfluidic mixing and nanoparticle determination (by light scattering and asymmetric Flow Field-Flow Fractionation), unilamellar vesicles with control on liposome size are easily generated [104]. On the other hand, lipids provide complete long-term passivation of nanochannel surfaces to a range of reagents, including proteins and nucleic acids. Passivation using lipid bilayers is significantly better than commonly used passivation protocols such as BSA, Casein and PEG [105,106]. Among other interesting developments in the field is lipid vesicle mediated transport of reactants as an alternative strategy to pressure-driven or electrokinetic flow-based transport in microfluidic devices. This strategy is based on the gliding of microtubules over channels coated with the molecular motor protein kinesin. The molecules of interest are loaded onto liposomes labeled with single-stranded DNA (ssDNA), and the liposomes are in turn loaded onto ssDNA labeled microtubules. The system allows for specific loading and unloading at the desired sites flagged with DNA molecules with a specific base pair sequence [107]. Another major challenge in microfluidics is handling and mixing ultrasmall volumes of reactants. Microfabricated silicon and plastic can provide reliable fluidic devices, but they are limited in the total volumes that they can handle (smaller than ~1 × 10^−12^ L). Recently, unilamellar lipid vesicles have been used to mix volumes as small as 1 × 10^−19^ L in a reproducible and highly parallelized fashion. In this strategy, vesicles made of lipids with opposite charges are fused together, leading to mixing of their contents [108].

**Figure 3:**
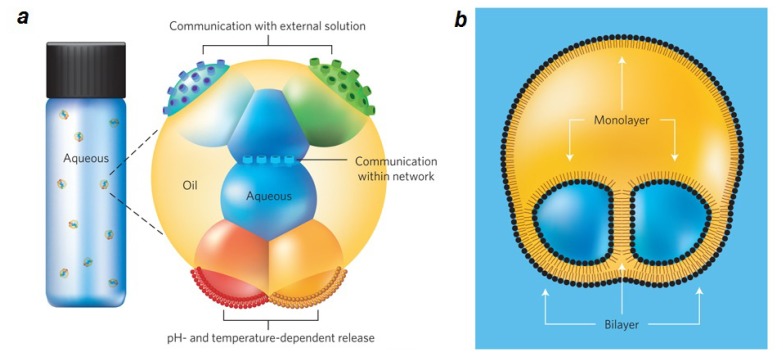

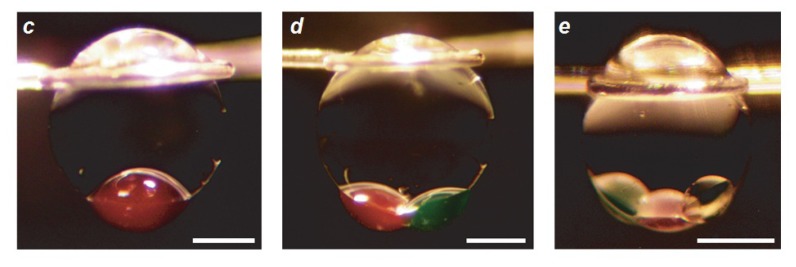
Network of lipid-encapsulated droplets. (**a**) Schematic illustration of a multisome. An oil drop encapsulates aqueous droplets that are connected by lipid bilayers. The bilayer incorporated protein pores allow the droplets in the network to communicate by exchanging molecules and ions. Similarly, pores in bilayers in contact with bulk solution provide routes for exchange between the droplet network and the bulk. Droplet content release can be triggered by pH- or temperature-induced rupture of the bilayers; (**b**) Schematic of an encapsulated two-droplet network, showing the lipid monolayers and bilayers. (**c**–**e**) Images of the multisomes containing one (**c**), two (**d**) and three (**e**) inner droplets. Oil drops were suspended on wire loops to allow extended analysis. Aqueous droplets were dyed with 25 μM sulphorhodamine 101 (red) or fluorescein (green). Scale bars, 400 μm. Figure is adapted from [112] with permission.

An important subfield of microfluidics is micro and nanodroplet engineering [109–111], in which the properties of lipid molecules have also been employed. Aqueous droplets in oil stabilized by a monolayer of lipids and joined through interface bilayers can be made. These droplets allow for engineering networks of droplets both in water and oil phases. Such a design is applicable to tissue mimics in medical applications and synthetic biology, but can also be applied to fabricate light sensors and batteries by incorporating pumps, channels and pores into the bilayers. Furthermore functional bilayer interface was formed in nanoliter size multisomes.—networks of aqueous droplets that are encapsulated within small drops of oil in water—that can be useful for drug delivery [112] (Figure 3). Droplet interface bilayers formed by contacting lipid monolayer-coated aqueous droplets against each other in oil–lipid media, can be permeabilized in a number of ways, allowing for triggered release of materials. One strategy is incorporation of photo-polymerizable phospholipids that crosslink upon UV irradiation. Crosslinking in turn results in pore formation across the bilayer and material transport [113].

Another interesting application of lipids in microfluidic systems is in generating nano-droplets with reduced size and increased stability compared to commercially available lipid emulsion with the size range of 200–300 nm which is too large to be used as tumor targeting. In this direction, lipid nano-emulsions (LNE) with 50 nm droplet size and 12 month stability have been designed and experimentally realized [114].

Finally phospholipids and phosphocholine derivatives have been proposed and used for passivation of surfaces [115,116], particularly in nano- and microfluidics [105]. Biofouling or biocontamination is relevant in a wide range of applications, including medical equipment and implants, biosensors, textiles, food packaging, water purification systems, and marine equipment [117]. Phosphorylcholine derivatives and PEG are two main categories of nonfouling materials that resist protein adsorption and cell adhesion [118]. Phosphatidylcholine helps to suppress nonspecific binding of cellular membranes, while accommodating specific functional binding of membrane proteins [119]. This property has been exploited in micro and nanofluidics extensively during the past years [75]. Supported lipid bilayers have been successfully generated and manipulated in microfluidic channels, for example using electrophoresis and shear flows [120]. The physicochemical properties of the bilayers have been modulated to enhance their anti-fouling capability while adding specific interaction sites [108,121].

### 2.3. Lipid Nanoassemblies

Lipid nanoassemblies have been designed and used in various fields including soft matter physics and synthetic biology where lipids are used to generate bio-mimetic systems [122]. Besides bilayer films, liposomes and micelles, lipids can also form lipid nanotubes (LNTs). LNTs can modulate the nucleation, growth, and deposition of inorganic substances both on their external surface and in their hollow core. The LNTs can thus be used as scaffolds for fabrication of 1-D nanostructures, including 1-D arrays of quantum dots and hybrid nanotubes consisting of functional inorganic nanoparticles embedded in the lipid membrane wall [123]. Lipid nanoassemblies can be manipulated and deformed optically and electrically in a controlled manner [124,125]. Lipid structures such as liposomes and tubes can enclose reagents and nanoparticles and can be glued to other nano-devices using a variety of strategies, including covalent linkages, bonds formed between biotin and members of the avidin family, and electrostatic gluing. Recently, “PC-inverse” choline phosphate (CP) was used to bind molecules onto a variety of cell membranes (e.g., Red Blood Cell and Hamster Ovary Cell) and on to PC-containing liposomes [126].

An interesting property of lipid vesicles is their passive remodeling capability when supported by elastic materials. Lipid bilayers are themselves usually fluid, do not support shear stress, and have a low lysis tension. However, when combined with an elastic material, the membrane acquires shear and tensile strength, while at the same time the bilayer allows for surface-area regulation. This dual form of mechano-protection is in fact an important mechanism by which animal cells are able to be mechanically strong yet highly deformable. Synthetic polymer-reinforced liposomes could find use as enhanced drug delivery vehicles. The membrane’s fluid character would enable the regulation of surface area and permeability, whereas the polymer support would provide a protective wrap [127].

Lipid structures have been assembled on solid state nanopores or combined with nanoparticles. By vesicle fusion, bilayers spanning micro- and nanometer sized wells have been generated [128]. Coating nanopores with a phospholipid bilayer tailors their surface chemical properties and allows fine-tuning and dynamic variation of pore diameters with subnanometer resolution [129,130]. Nanoparticle-vesicle hybrids can also be constructed, in which particles reside between the leaflets [131]. Such structures can be used for medical purposes such as triggered drug release. When superparamagnetic iron oxide nanoparticles-vesicle hybrids were exposed to an alternating magnetic field, the particles locally heated up, resulting in spatially and temporally controlled release of drugs [33]. Another interesting hybrid design is glass nanopipette-supported bilayers, which provide a highly stable lipid bilayer (comparable to the plasma membrane of cells) for single-molecule studies. The structure is stable for hours at 900 mV, allowing for single protein channel studies by patch clamping [132].

Lipid bilayers have also been extensively used to solubilize carbon nanotubes. Carbon nanotubes (CNTs) are quasi-one dimensional cylindrical carbon nanomaterials that have been considered as nano-containers for drug delivery and as nano-injectors for drug passage through lipid membranes. One major drawback associated with single-walled carbon nanotubes (SWNTs) in aqueous environments is their propensity for aggregation, which severely limits their application. Several studies showed that phospholipids can solubilize SWNTs and provide a solubility superior to that provided by nucleic acids, proteins, or detergents [133,134]. Carbon nanotube-lipid assemblies were first fabricated in the early 2000s via self-organization [135]. The assemblies were then purified by application of an electric field to produce monodisperse, water-soluble CNT-lipid assemblies [136].

Lipids have also been used to engineer soluble hybrid nanoassemblies in combination with molecules that are insoluble in aqueous buffers. A prominent example is provided by nanodiscs: discoidal phospholipid bilayers (typically 10–12 nm in diameter) enveloped by a stabilizing amphipatic helical membrane scaffold protein (apolipoprotein A-I derivatives) [137]. Monodisperse (with controllable size), stable and soluble nanodiscs with surface immobilization capability have been engineered by self-assembly. Lipid nanodiscs have found applications in various areas. Most notably trans-membrane proteins were integrated into the bilayer part of lipid nanodiscs, allowing for biophysical studies of the reconstituted proteins [138–143]. Lipid nanodiscs have the capability of harboring hydrophobic or amphipathic molecules (e.g., drugs) and thus can serve as a nanocontainer. Nanodisc containers can be used for drug delivery and also for magnetic resonance imaging, where nanodiscs loaded with amphiphilic chelators of contrast agents are used for contrast enhancement [144,145]. Furthermore, nanodiscs containing rhodopsins are attracting attention for their potential use in photonic applications [146].

### 2.4. Lipid Nanoelectronics and Photonics

The term “nanoelectronics” refer to the use of nanotechnology to develop miniaturized electronic components to be applied in information processing, telecommunications and signal processing [147,148]. Biological systems are inherently designed to handle material and information transport on a small scale in a crowded cellular interior. Biological design principles as well as building blocks can be incorporated into nanoelectronic devices to enable new generations of electronic circuits that use biomimetics to carry out complex tasks. For instance, by inserting proteins in a lipid bilayer shell covering the nanotube or nanowire, ion channels and pumps can be integrated into single-walled carbon nanotube (CNT) and silicon nanowire (SiNW) transistor devices. These hybrid devices have been used for example to couple biological transport to electronic signaling [149]. Lipid bilayers with embedded protein pores have also been used to construct a light sensor, a battery, and half- and full-wave rectifiers [150,151]. One important issue is the scalability of such designs. Recently lipid monolayer-coated hydrogel shapes (e.g., sphere, cube, hexagon, crescent and cross) have been proposed as building blocks for constructing scalable nano-microelectronic structures and nano-micromechanical devices such as switches, magnetic field driven rotors and painted circuits [152].

Lipids have also been interfaced with graphene, a single layer of carbon atoms in a honeycomb lattice with exceptional physical properties that make it a promising material for a wide range of applications, particularly in electronic devices [153]. The manipulation of graphene’s physical and chemical properties is therefore a growing area of research. Its electronic properties can be, for example, modulated by a charged lipid bilayer adsorbing on its surface. This technique has been used to fabricate an electrolyte-gated biomimetic membrane graphene transistor. The device has been applied to electrically monitor the interaction of peptides with the bilayer [154]. When exposed to graphene sheets, anionic liposomes spontaneously reorganize into a stable lipid monolayer covering the surface of the graphene [155]. Graphene oxides (graphene with OH and COOH functional groups) have also been combined with lipid bilayers to make hybrid structures including alternating supported lipid membrane and graphene oxide layers [156].

Another widely explored area of research is fabrication of hollow cylindrical lipid tubules filled with various optical and magnetic materials. Examples include lipid tubules containing magnetic rods or gold nanowires assembled from nanoparticles [158,159]. An interesting development was to fabricate optically anisotropic fibers of various shapes by filling the hollow core of cylindrical lipid tubules with nematic liquid crystals through capillary action. The tubules were then aligned on a glass substrate to form 2D ordered arrays of tubules with birefringent cores [160].

**Figure 4:**
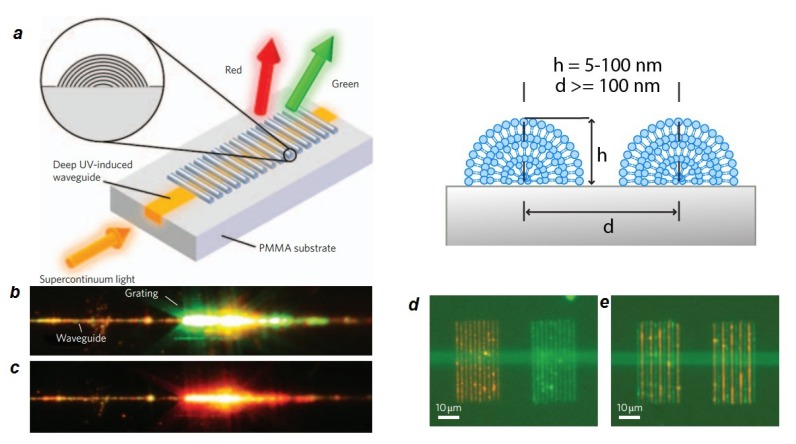
Lipid multilayer optical diffraction grating. (**a**) Schematic of the functional waveguide grating couplers. Light of a supercontinuum laser source is coupled into a single-mode strip waveguide through an optical fiber and decoupled under different angles by the grating coupler; (**b**,**c**) Photographs of the coupler at 308 and 458, where the red and green portions of the guided laser are coupled to radiation modes, respectively; (**d**,**e**) Fluorescence overlay of red and green fluorescence from rhodamine and fluorescein labelled lipids, respectively, integrated with a pitch (=2d) of 2 μm by dip-pen nanolithography (DPN) patterning on a waveguide (horizontal green line is due to autofluorescence). Figure is adapted from [157] with permission.

Finally, lipids have also been used to create multilayer structures with useful optical properties. One interesting design (Figure 4) is a multilayer optical diffraction grating [157], printed by dip-pen nanolithography (DPN) of biofunctional lipid multilayers with controllable heights between ~5 and 100 nm and period of larger than 100 nm. DPN is a unique technique in having a constructive and parallel nature that enables integration of multiple materials on structured surfaces that have been pre-fabricated by top-down lithographic methods. The size and shape of the grating can be modulated by analyte binding, which is a property that allows for biosensing applications. Nanostructured lipid multilayer arrays have been developed by combining microcontact printing with nanoimprint lithography and DPN [161]. This method is capable of producing nanostructured lipid multilayer arrays with capability in lateral patterning and scalability, high control on topology, and bottom-up integration of a variety of biological materials such as lipids with high resolution (nanometers).

## 3. Computational Lipid Nanotechnology

The steady increase in computational power along with progress in methodologies has allowed the study of lipid nano-assemblies. Box II provides a quick overview of the most advanced simulation methodologies, each of them providing an appropriate platform for studying processes at different time and length scales. We then briefly discuss the current status of the membrane simulation research field with a particular focus on lipid-based nanostructures, material transport at the nanoscale mediated by membranous structures, and interaction of nano-devices with lipids and lipid membranes.

BOX II **Advances in lipid simulations**

**(1) Force-fields**

A force field is a set of parameters and mathematical functions describing the potential energy of a system of atoms and molecules. The force field is used to simulate the structure and dynamics of lipid systems. Depending on the requirements of specific applications, different levels of accuracy are considered in the definition of a force field. In the category of atomistic force fields, ‘All-atom’ force fields provide exact parameters for every single atom including hydrogens, while the ‘united-atom’ force fields consider only the carbon and hydrogens in methyl and methylene groups, considering each as a single particle. These approaches, though very accurate, can only be used to simulate small lipid systems for short time scales. For simulations with larger size and time scales, a number of ‘coarse-grained’ force fields have been introduced. Experimental measurements and very accurate quantum chemical calculation are used to derive force field parameters.

**(2) Atomistic force fields**

The CHARMM force field, which explicitly describes all atoms including hydrogen atoms, is the most widely used all-atom force field [172] and is considered as the most accurate method to simulate lipid membranes. CHARMM parameters for lipids were obtained on the basis of ‘*ab initio*’ calculations [173] (within the Charmm22 parameter set, often denoted as C22). This force field benefits from a broad range of parameters for dihedral angles as well as Urey-Bradley term for covalent angles. Modified versions were published later in [174] (Charmm27, or C27 parameter set) [175], (C27r parameter set) and finally in [176] (C36 parameter set). As a result of all these reparameterizations, many discrepancies with experimental results were eliminated [177–179]. However, using either TIP3P or SPC water models could improve the results for area per lipid, bond order parameters, electron density and the structure factor [179,180].

Although performing calculations using all-atom force fields gives the best accuracy, they are often computationally inefficient. United-atom models are in principle less accurate, but are often more practical [181]. Among the united-atom force fields, GROMOS [182] has reportedly the best performance. In this approach aliphatic chains of carbon and hydrogen are presented as a united interaction center, which makes these simulations three times faster than all-atomic simulations. Various versions of the GROMOS force field have been already established. In the most recent one, in addition to modified non-bonded interactions [182], the Ryckaert-Bellemans potential was implemented to describe torsion rotations of the hydrocarbon tails of lipids. The GROMOS force field is fully supported in the GROMACS simulation package [183]. Many early simulations of lipid membranes were carried out with the Amber force field [11,14]. In the initial versions of this method the average area per lipid was underestimated [184,185]. This is even the case in the modified version known as GAFF force field [186]. It appears that the GAFF force field needs further optimization to reproduce correct bilayer structures for a tensionless membrane.

**(3) Coarse-grained force fields**

Many biochemical phenomena happen over such long time and large length scales that all-atom approaches are not required to describe the system. Examples are thermal undulations of the membrane, self-assembly into micelles, vesicles, lamellar or hexagonal phase transformations, and lipid domain formation. In these cases, to construct a much simpler but less accurate method, a group of atoms is replaced with pseudo-atoms, leading to a coarse-grained (CG) and a mesoscopic description of a system of lipids. The coarse-grained force fields vary in their protocols for grouping atoms and their interaction potentials, as well as the solvent models used. Water effects can be modeled by applying either explicit coarse-grained pseudo-atoms or implicit solvents, which are described by effective potentials. In the CG approaches, the potentials describing the interactions between the particles are effectively derived through atomistic simulations using inverse Monte-Carlo (MC) methods [187], inverse Boltzmann [30,188] methods, or thermodynamic calibrations of data [35,189]. Some quantities used to parameterize the intramolecular potentials include the radial distribution functions and distributions of intramolecular distances.

One of the most successful coarse-grained models is based on the Martini force field, which uses an explicit solvent [189][190]. In the Martini force field, four heavy atoms are replaced with one pseudo-atom. The interaction potential consists of Lennard-Jones, bond-length, bond-angle and also electrostatic terms, which are tuned to reproduce experimental partitioning data. During the last few years the Martini force field has been used widely to study many different phenomena in lipid membrane especially in modeling the lipid-mediated nanoparticle reactions. In most cases the results of these simulations agreed well with experiments. Some studies involve formation of lipid domains and rafts due to the spontaneous separation of liquid-ordered and liquid-disordered phases [191], monolayer collapse [192], lipid self-assembly and vesicle formation [31,35,36,193], flip-flop motions across the lipid membrane [194], nanoparticle transport and accumulation in lipid bilayers [195], spontaneous curvature and stability of asymmetric bilayers [196], membrane curvature and lipid packing [197] and voltage-sensitive dyes with a lipid membrane [198]. Freezing the coarse-grained water in the room temperature is one of the main disadvantages of the Martini force field. However this problem can be tackled by introducing artificial ‘antifreeze’ particles [190,199,200].

A more precise coarse-grained lipid model with explicit water has been introduced and used in [200,201] and [202]. In this model ellipsoidal particles are used to coarse-grained the lipid particles. Each water molecule is considered as a spherical particle with a dipole. The interaction between particles was defined by an effective Gay-Berne potential with charges embedded on the head group and dipoles at the ester groups. This model is an appropriate force field to study the properties of PC bilayers including membrane electrostatics, pressure distributions, spontaneous curvature and water permeation [202]. Dissipative particle dynamics (DPD) is a CG technique in which an efficient thermostat is provided by dissipative and random pairwise forces, which act together with short-ranged conservative soft repulsive forces [203]. DPD has been successfully employed to simulate the equilibrium structure and elastic properties of fluid bilayer membranes as well as dynamic membrane processes such as fusion [204]. A main disadvantage of the method is a lack of a direct link between the model parameters and real molecular parameters. Typically some experimental observables are used as criteria to set the simulation parameters [205]. The simulation results were consequently only partly in agreement with experiments. For example, in earlier parameterization schemes the simulated lipid bilayers exhibited a reasonable equilibrium structure in the absence of stretching. However upon stretching, the bilayer ruptured at 60% stretching [206], while rupture occurs after 5% stretching in experiments [207]. This discrepancy has been improved recently, but the problems are not fully resolved yet [203]. Nevertheless, DPD is widely used in soft and lipid nanotechnology in view of its computational efficiency [208–212].

### 3.1. Lipid-Based Nano-Structures *in Silico*

#### Flat and Curved Bilayers: Nano-Vesicles and Tubes

Bilayer with different phase behaviors—fluid, gel, ripple and interdigitated—have been subject to simulation studies [162,163]. Furthermore, bilayers with a range of geometries and topologies have been constructed *in silico* and characterized. For constructing these structures, it is required to generate curved and/or twisted bilayers. A key parameter that controls the preferred shape of a lipid assembly is the packing parameter of the lipids, defined as [164],

$$P=\frac{V_{t}}{l_{t}A_{h}}$$

where *V_t_* and *l_t_* are the volume and length of the hydrophobic tail and *A_h_* is the average area per head group. Spherical micelles form when $P\leq \frac{1}{3}$, whereas for cylindrical micelles one expects $\frac{1}{3}\leq P\leq \frac{1}{2}$, for flat bilayers $\frac{1}{2}\leq P\leq 1$, and for inverse micelles *P* > 1 [164].

It has been shown by simulations that the lipid shape accompanied with the membrane curvature can produce a sorting mechanism. Lipids with a larger head group (positive preferred curvature) tend to localize in the outer leaflet of a curved bilayer, while the opposite is true for lipids with smaller head groups (negative preferred curvature). This sorting phenomenon is especially important when the mean curvature is very high, such as in nano-vesicles and nano-tubules with a radius of the order of 10 nm [165]. In cells, other physico-chemical properties such as lipid phase transitions or interactions with peripheral or integral membrane proteins can also influence lipid sorting. Membrane curvature can cause phase segregation which results in soft and rigid domains. Here, soft domains prefer the strongly curved areas, whereas the rigid domains move toward the flat regions [166]. Therefore, bending rigidity is the other controlling parameter for lipid sorting coupled to membrane curvature [167,168].

Cholesterol is known for its role as a modulator of the physical properties and lateral organization of the plasma membrane lipid bilayer. One of its major roles is reported to be the broadening and eventual elimination of the cooperative gel to liquid-crystalline phase transition (L_β_→L_α_) and its replacement by a phase with an intermediate degree of organization. When lipid sorting is driven by bending rigidity, the role of cholesterol in modulating the membrane phase properties becomes more important [169]. Evidence for this effect based on analysis of the influence of cholesterol on producing phase separation in a lipid-cholesterol mixture was studied [170]. Further, the density of cholesterol was found to be larger in the high curvature region in a membrane composed of brominated di 18:0 PC/cholesterol 2:1 [171].

**Figure 5:**
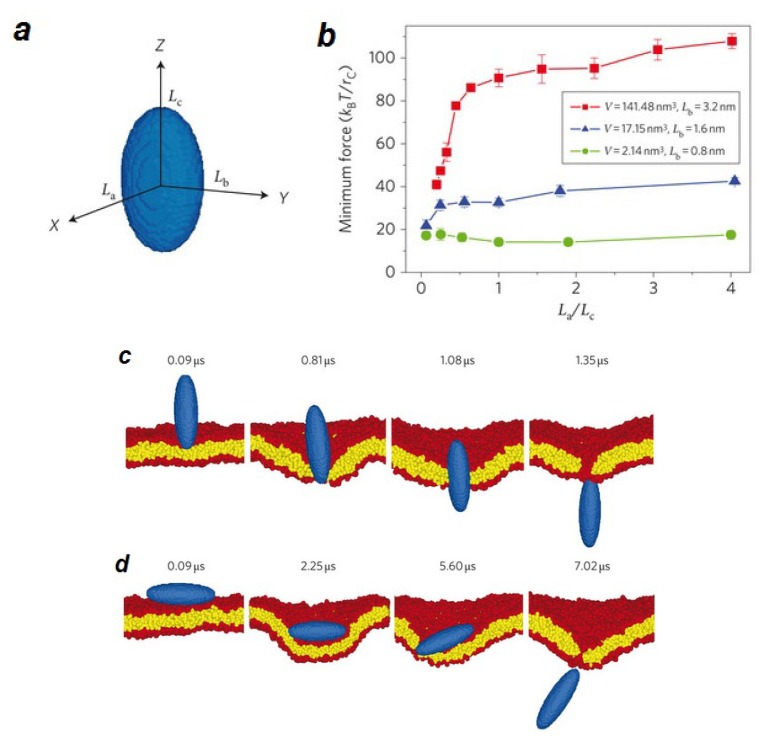
Computer simulation of the translocation of nanoparticles with different shapes across a lipid bilayer (**a**), an ellipsoid particle is schematically illustrated. (**b**) The minimum driving forces needed to pass the ellipsoid particles of different volumes through the lipid bilayer. The shape anisotropy of the nanoparticles is adjusted by changing the aspect ratio (*L*_a_/*L*_c_) at fixed *L*_b_ and volume. r_c_ is the force range. (**c**,**d**) Snapshots of computer-simulation of the translocation of the particle with vertical (**c**) and horizontal (**d**) initial orientations. *L*_a_ = 1.6 nm, *L*_b_ = 3.2 nm and *L*_c_ = 6.4 nm (**c**); *L*_a_ = 6.4 nm, *L*_b_ = 3.2 nm and *L*_c_ = 1.6 nm (**d**). Blue, ellipsoid; red, lipid heads; yellow, lipid tails. Figure is adapted from [214] with permission.

Integral membrane proteins can induce a local deformation in the plasma membrane [213]. By performing computational studies, Sperotto *et al.* found that the hydrophobic mismatch between the protein and the lipid bilayer controls the local deformation of bilayer. On the other hand, nanoparticles deform the bilayer locally when they translocate across the cell membrane [214] (Figure 5). The size of the disruption area due to the penetration of the nanoparticle is a function of the size, shape, orientation of the particle and also of the local curvature of the bilayer. A disruption area larger than 2 nm^2^ leads to a strong deformation of the lipid bilayer and consequently a transient pore forms across the membrane. Pores facilitate translocation of drugs, ions, lipids and proteins through the membrane.

### 3.2. Membrane-Mediated Transport at the Nano Scale

#### 3.2.1. Lipidic Nano-Pores and Pore-Induced Transport in Lipid Membranes

To release materials that are enclosed within a bilayer, one strategy is to induce nano-pores/ruptures within the bilayer allowing for material transport such as ion permeation [215] or lipid translocation [216]. Since these pores are transient, a “lipidic gate” can be opened and closed at a desired time. The transient nature of lipid nano-pores has made it difficult to precisely study their structure by experiments; thus lots of effort has been made recently to investigate the origins, properties and functionality of pores by computer simulations both at the atomistic and coarse-grained levels [217,218].

Although pores can form spontaneously, various operations enforce this procedure. Here some of the most important pore-inducing agents and conditions are briefly mentioned.

- *Electric field poration.* One of the best-known ways to create pores is by exerting electric potential differences to the membrane of a cell. Experimentally, electric pulses produced by high-energy lasers are applied to the cell membrane. Computationally, the proper potential difference across a membrane is produced either by applying a constant electric field [219–227], or by creating an ionic imbalance around the membrane, which causes an electric field responsible for pore formation [227–230]. As a result of the electric field, water molecules can penetrate into the membrane. The process of opening a pore usually starts by spreading of water molecules throughout the membrane. When water molecules permeate the membrane, some small water defects form around the lipid head groups. A pore will be later stabilized when the head groups move towards this initial pore. The pore then enlarges and becomes lined with lipid head groups and the head groups distribute evenly over the pore “surface” [218].
- *Pore formation induced by mechanical stress*. Mechanical stress such as osmotic swelling can also induce pore formation in lipid bilayer membranes. In this case pores are formed as a response of the membrane to the surface tension. To simulate a membrane under different levels of surface tension, one possibility is to change the surface density of lipids. Forming a pore requires a critical threshold surface strain, which depends on the lipid composition and environmental condition [189,218]. By estimating the free energy of the system, the stability of pores can be investigated. Several ways have been proposed to calculate free energy differences in membrane systems caused by pore formation. One possibility is to use constraint forces to calculate the free energy profile for pore formation as a function of pore radius [231–233]. Another option is to calculate the free energy of pore evolution by pushing a lipid molecule into the bilayer interior in a reversible manner [233]. During this process, a small water pore forms spontaneously.
- *Pore formation in lipid membranes by peptides*. One of the main pharmacological mechanisms for killing microbial cells is by antimicrobial peptides, which form pores in the bacterial cell membrane, leading to ion influx and cell death. Typically, amphipathic peptides containing lysine and arginine residues are able to induce pores. Pore formation occurs when the hydrophobic parts of the peptide are in contact with the lipid membrane and the polar residues face the water. Usually, formation of a toroidal membrane-spanning pore is the result of the cooperative self-assembly of five to six magainin (an antimicrobial peptide) molecules [163,218]. The first computational study of peptide-induced pores suggests that pores can be formed in a few nanoseconds [234,235]. Recent molecular dynamics simulation studies reported also a time scale between 10 to 100 nanoseconds for pore formation in the presence of enough peptide [236].

Pore formation is usually accompanied by ion transport across the membrane, especially when an ionic imbalance is the forcing factor to open a pore. Cations like sodium permeate the bilayer primarily through water pore defects, while for anions like chloride ions, the pore-mediated permeation is extremely unlikely. A thin water chain is enough for the cations to diffuse across the membrane with a mechanism similar to proton diffusion along a water chain. This is not possible for negative ions. Obviously large pores (>1.5 nm) can readily support the flux of anions [230,237]. The rate of ion permeation through intact bilayers can be estimated by calculating the free energy barrier of this process. Membrane permeability rates of sodium ions are calculated to take in the order of a day, which is in good agreement with experimental estimations [233]. Therefore, membrane nano-pores dramatically increase the membrane permeability for ions.

Lipid flip-flop is a biological phenomenon during which one lipid or cholesterol is displaced from one leaflet to the other. Many computational studies have proposed that transient pores are the most important pathways for passive phospholipid flip-flop across the membrane [236,238,239], a process that happens over a nanosecond time scale. In the absence of pores, the time scale for flip flop would be on the order of hours. Simulation studies showed that cholesterol does not need pores to flip-flop and its flip-flop rates depend on the composition of the bilayer [240]. A cholesterol molecule can flip across the bilayer within a few milliseconds. It was shown computationally that the free energy barrier for translocation of one cholesterol molecule in a DPPC membrane is about 24 kJ/mol, much larger than the barrier for DPPC flip flop, which was about 78 kJ/mol [218,233].

#### 3.2.2. Fusion and Fission of Lipid Membranes

In some applications such as drug delivery and microfluidics, nano-vesicles are used as vehicles to transfer materials to another membrane-enclosed compartment such as a cell or another vesicle (e.g., containing a second reactant). The final step in this process involves membrane fusion, which is also a key process in cell biology [218,241]. Fusion is still poorly understood, since many lipid and protein molecules contribute in a multi-stage mechanism, complicated deformations of the membranes occur, and entropic effects also contribute. In the past few years, many computational efforts were made to investigate the role of specific molecules and to study hypothetical mechanisms required for membrane fusion [242–248]. The first step of the fusion process is forming a stalk along the region of possible contact between two membranes, then by expansion of the stalk two inner monolayers contact to form a hemifusion diaphragm. The procedure completes when a pore forms in the hemifusion diaphragm [241–243]. The temperature and the spontaneous curvature of the lipids affect the expansion of the stalk [249,250]. The distance between two approaching membranes influences whether they can form a link [218]. It was shown that a minimum distance of 2–3 nm is essential to form a bridge between two adjacent membranes [241,251]. The formation of the stalk critically depends on the hydration state of the headgroups of the two opposing bilayers [246,252,253]. The lipid composition also impacts the rate of stalk formation [243,254]. Moreover, the lipid tail length and rigidity influence the fusion as well. Lipids with shorter or stiffer tails prevent the formation of a stalk since they have difficulty undergoing conformational changes [248,255,256]. Although many studies have addressed membrane fusion, many issues still remain elusive [241]. Challenges are for instance the cooperative behavior of lipid compositions [218,243,252,255,256], the effect of different morphologies of membranes such as planar bilayers *versus* vesicles [249,251,252,255,256], and also the stalk evolution in SNARE mediated fusion [241].

Fission and budding are other examples of biological processes in cells that require deformation of membranes and are also involved in material transport. These processes occur during exo- and endocytosis, intracellular trafficking, cell division of budding yeast and enveloped virus infection [218]. Budding and fission are processes in which multiple separate cells are generated by cell subdivision. As in the case of fusion, many factors contribute to membrane deformation leading to the fission events. Some of these driving forces have been studied experimentally and computationally and their roles have been proved. Some of them are; (i) the interfacial energy or the line tensions due to the existence of different separate domains in lipid membrane [257–265], (ii) the asymmetry of the two leaflets of the membrane, caused by different lipid compositions and by different interactions of the two leaflets with peripheral membrane proteins such as the cytoskeleton or the glycocalix [266–272], and (iii) the attractive or adhesive interaction between the nanoparticle and the lipid membrane [273].

### 3.3. Lipid-Nano Device Interaction Studies

A large part of the literature on *in-silico* lipid nanotechnology is focused on investigation of nano-objects such as carbon nanotubes, fullerenes, graphene, and metallic nanoparticles with lipid bilayers (Table 1). Carbon-nanotubes have high surface area, which allows them to carry significant amounts of drugs, proteins and DNA molecules. CNTs show adsorption and photoluminescence at near IR, which can be used to image their location and to destroy nearby cellular structures [274,275]. CNTs likely enter the cytosol by passage through the lipid bilayer or via endocytosis, but the molecular mechanism of CNT uptake is not yet understood. A recent all-atom molecular dynamic simulation confirmed that nanotubes can pass the bilayer in a process that involves multiple steps [276]: landing and floating, penetration of the lipid headgroup area and finally sliding into the membrane core.

Another interesting application of CNTs is as nano-injectors. CNTs have been thought of as needles that can penetrate the bilayer and inject materials via their hollow inner spaces. Thus CNTs can potentially be used to inject materials into single cells. This process has been studied recently by computational nanotechnologists and the energetics of the process and potential clogging of the CNT by the lipids, as well as conversely possible perturbation of lipid bilayer by CNTs have been addressed. In one study, using coarse-grained molecular dynamics simulations, penetration of the membrane by single-wall CNTs led to the association of lipids with the periphery of CNTs as well as lipid entrance into the inner space of the tube. The study showed that the lipids spread out within the inner space and can block the tube. Fortunately the blockade can be avoided with fast penetration [277]. The free energies associated with bilayer rupture during CNT penetration were investigated using non-equilibrium, all-atom molecular dynamics simulations for POPC bilayers and found to be within the estimates of rupture by an electric pulse [278]. The forces of penetration depend on penetration velocity and were overestimated in nonequilibrium all-atom molecular dynamics simulations as compared to experimental values. After penetration, the presence of the CNT restrains the conformational freedom of neighboring lipids and thereby impacts the membrane stabilization dynamics [279].

Due to the medical importance of the fullerene family [280], and especially C60, computational efforts have been devoted to study the interplay between fullerenes and lipid bilayers. Fullerenes are particularly interesting for the delivery of drugs such as cationic peptides. Molecular dynamics simulations have addressed the transport efficiency of cationic species across lipid membranes. It turned out that fullerene readily takes up cationic molecules, but the more positive the groups are conjugated, the more hydrophilic the nanostructure will become, and consequently the efficiency of bilayer penetration declines [281]. Penetration of fullerenes into lipid bilayers has been found to be a spontaneous process irrespective of the size of the fullerenes (C60, C180, C540) [282]. Fullerene molecules have a high propensity for aggregation in aqueous solution. However they disaggregate after entering the bilayer interior in a process that occurs spontaneously on microsecond timescales [283]. As in the case of CNTs, the penetration of lipid bilayers by fullerenes leads to bilayer distortion [282]. Graphene was observed to remain stable in between the leaflets in the hydrophobic core of the bilayer [284]. This allows for coating of graphene layers with phospholipids, which in turn allows for further addition of bio-functionalities.

The interactions of nanoparticles such as carbon or metallic nanoparticles with membranes are governed by the size, shape, charge and hydrophobicity of the particles [189,212,285]. For example, small carbon nanoparticles (C4) enhance the probability of water penetration through the bilayer and cannot disrupt the bilayer whereas large particles (e.g., C45) induce rupture of the bilayer [286]. Shape is also an important determinant. Physical translocation of nanoparticles with different geometries (e.g., sphere, ellipsoid, rod and disc) has been studied computationally. Shape anisotropy as well as the initial orientation of the particles with respect to the membrane determines the translocation capability. Rotation of the particles during penetration complicates the penetration process [214]. Two rotation processes can be recognized: first rotation occurs when the particle comes into contact with the bilayer because of the resistance of the lipid bilayer. The second rotation occurs during the penetration when the lipid bilayer is significantly deformed by the embedded particle. The particle is enforced—by the driving force and the elastic response of the bilayer—to rotate in order to orient its long axis as perpendicular as possible to the plane of the membrane. The simulation studies showed that the first rotation hinders particle penetration whereas the second one assists penetration. Electrostatic forces are also determinants of lipid-nanoparticle interactions. In a systematic coarse-grained molecular dynamics simulation, it was shown that binding of nanoparticles to membranes is facilitated by electrostatic interactions. High electrostatic energy can even lead to the wrapping of the membrane around charged nanoparticles [41]. For gold nanoparticles it was found that functionalized particles with cationic groups penetrate into the bilayer and disrupt it. Reduction of cationic groups can eliminate hole formation in physiological conditions [287]. Finally the effect of the hydrophobicity of penetrating nanoparticles on the lipid bilayers has been studied computationally, and it was found that the particles enter the hydrophobic core of the membrane in a process which is driven both enthalpically and entropically [288]. Amphiphilic ligands facilitate nanoparticle penetration. This effect depends on the rigidity, size, orientation, density and anisotropic distribution of the ligands [189,205,285].

## 4. Concluding Remarks

Lipid nano-devices have the advantage of being biologically inert while specific functionality can be added by inserting proteins and sugars into the bilayer. In medical applications, lipid nanodevices can be designed to exploit the efficient communication strategies that cells use to interact or that enveloped viruses use to infect cells. A major advantage of lipid nano-devices in medical, mechanical and chemical engineering is their ability to form nano-sized containers by self-assembly. The containers can be triggered to release their content at a desired place and time in a reversible manner due to self-healing of the induced lipid nano-pores. Lipids have proven useful as stabilizers of other nano- and micro-structures such as droplets and nano-particles. It has been shown that the interfacial lipids can do more than being a stabilizer and an enclosure. Recently interfacial lipids have been used to regulate localized activity of DNA molecules in microdroplets [298,299]. The DNA molecule can adopt two conformations, extended coil conformation when it interacts with phospholipids (and Mg^2+^), and condensed conformation in the presence of the polyamine spermine. The extended conformation turns on the transcription machinery and consequently the localized production of RNA is triggered. An additional interesting property of phospholipids is their boundary lubrication. Lipids also enhance the efficiency of other lubricants such as hyaluronan, which is of interest to surface scientists and biomedical engineers [300]. Lipid nanoelectronics and photonics are in their early stage, where fundamental aspects of lipid-metal and semiconductor interactions are being investigated.

Some lipid nanotechnology products are in the industrial phase and entering the market. In microfluidics, lipid technology has already been integrated into lab-on-a-chip platforms to passivate surfaces, stabilize droplets and transport materials. In medicine and biosensing, lipid nanotechnology has already found a large number of applications and several lipid-nanodevices have secured FDA approval for clinical trials. In the cosmetic and food industry, the significance of lipid nanotechnology is increasingly appreciated. Food manufacturers—particularly those involved in the oil and fat industry—can gain a more competitive position through the application of lipid nanotechnology, which allows them to design safe and healthy food with improved functionality and performance.

With the advances in computational physics and chemistry and in particular coarse-graining strategies, modeling nanoscale phospholipid assemblies has become feasible. Simulations have provided prior knowledge to experimentalists, and guided them towards the most interesting designs. The help of simulation studies is valuable, particularly, as the experiments are performed at small scale and sometimes at the level of single nano-objects, where signal to noise ratios are low and a prior knowledge of the signal might be very helpful.

A fascinating direction for the future is designing new lipid molecules by adding new functionalities to natural lipids. Initial efforts in this direction have shown great promise. Charge switchable phospholipids have been designed and applied to gene delivery into cells [301]. Azo-benzene-based photo-switchable lipids have been synthesized that undergo cis-trans isomerization upon exposure to an electromagnetic wave in a wavelength-dependent manner. The conformational modulation allows for reversible control of the surface pressure and the molecular ordering of the lipids in assembled monolayers [302]. Furthermore, photo and thermo responsive azobenzene-containing lipids with gelation ability and supra molecular chirality have been designed and experimentally analyzed successfully [303]. Switchable amphiphilic molecules are of broad technological interest, specifically for the chemical industry and in environmental science [304].

## Figures and Tables

**Table 1:** Molecular dynamics simulations of bilayer based hybrid nanostructures.

Nano-object	Size of the nano-object	Lipid	Lipid structure	Simulation time	Force field/Software	Water model	Ref
Gold Nanoparticle	Radius of gyration:11.45 Å, Gold core:11.13 Å	DPPC/DPPG (3:1)	Bilayer	40 ns	MARTINI	P_4_	[289]
Fullerene	1 nm	Pure DOPC, Pure DPPC	Bilayer	88 μs	MARTINI	P_4_	[290]
C_180_, C_60_ C_20_	1.2, 0.72, 0.4 nm	Pure DPPC, Pure DLPC, Pure DSPC	Bilayer	800 ns	MARTINI	P_4_	[286]
C_60_, C_68_H_29_	1.07 ×1.1 nm^2^	DMPC/Cholesterol (3:1)	Bilayer	1.1 ps	DL_POLY 2.17 GUI [291], UA-OPLS [292,293] GROMACS v.3.3.1 and v. 4.0.5	TIP3P	[294]
Graphene	5.9 × 6.2 nm^2^	POPC	Bilayer	516 ns	MARTINI	P_4_	[284]
Graphene	2.425 × 2.380 nm^2^	CRPC	PC-Plate	10 ns	GROMOS	SPC/E	[295]
CNT	*L* = 2.35 nm, *R* = 1.87 nm	DMPC	Bilayer	1.58ns	AMBER 96	TIP3P	[296]
Hydrophobic NP	10 nm	DPPC	Bilayer	20 ns	MARTINI	P_4_	[42]
Semihydrophilic NP	10nm	DPPC	Bilayer	15 ns	MARTINI	P_4_	[42]
CG NP	6.8 nm	DPPC	Bilayer	320 ns	MARTINI	P_4_	[41]
CNT	*L* = 20 Å, *R* = 10 Å	Pure POPC, POPC/Cholesterol (7:3)	Bilayer	6 ns	CHARMM	TIP3	[278]
DWCNTs	*L* =33 Å, *R* =3.4Å	DMPC	Monomer	3 ns	CHARM27	TIP3	[297]
